# Experimental Research on Bonded Anchorage of Carbon Fiber Reinforced Polymer Prestressed Strands

**DOI:** 10.3390/polym14194015

**Published:** 2022-09-25

**Authors:** Liqiang Jia, Bo Wang, T. Tafsirojjaman

**Affiliations:** 1 Guangxi Xingang Communications Investment Group Corporation Ltd., Chenguang Road 100, Qinzhou 535008, China; 2 The Key Laboratory of Urban Security and Disaster Engineering of Ministry of Education, Beijing University of Technology, Pingleyuan Road 100, Beijing 100124, China; 3 CCCC Highway Bridges National Engineering Research Centre Co., Ltd., Deshengmenwai Street 85, Beijing 100120, China; 4 School of Civil, Environmental and Mining Engineering, The University of Adelaide, Adelaide 5005, Australia

**Keywords:** bond type anchorage, CFRP prestressed strand, bonding medium, anchoring efficiency

## Abstract

Aiming at the problems of a large number of corrosion and fatigue damage of the current prestressed steel strands, this paper adopts carbon fiber-reinforced composite (CFRP) strand with better corrosion resistance and fatigue resistance and uses it in concrete structures. The bond anchorage is usually used to anchor CFRP tension members, which bonds the CFRP through the binding medium. Through experimental research on the CFRP strand bond anchorage, the inner taper of the CFRP prestressed strand cone was anchored and the influence of different anchor lengths and bonding media on the anchorage performance was determined. The test results demonstrate that the taper of the conical anchorage described in this paper is a key factor affecting its anchorage performance and increasing the inner taper within a certain range is beneficial to improving the anchorage performance of the conical anchorage. The bonded anchorage of the CFRP prestressed strand with a 200 mm anchor is the most reliable and efficient, as the taper of the 200 mm anchor is the largest. The average anchoring efficiency coefficient of the 200 mm anchor was 96.4%, which is 3.7% and 2.6% higher than the average anchoring efficiency coefficient of 220 mm and 250 mm anchors, respectively. The anchoring efficiency of the anchor is also high (94.1%) when the epoxy resin mortar is used as the bonding medium. Moreover, after an appropriate amount of quartz sand is added to the epoxy resin, the overall comprehensive performance of the anchor can be improved to a certain extent and the stress of the CFRP strand can be improved. The coupling between ultra-high-performance concrete dry mix (UHPC-GJL) and CFRP strand materials is not suitable for UHPC-GJL being used, as its binding medium as the average anchoring efficiency coefficient is only 44.5% when UHPC-GJL is used as the anchor bonding medium.

## 1. Introduction

Bridges have sprung up significantly in people’s field of vision due to the enhancement of the world economy and the rapid development of infrastructures, making great contributions to the world’s economic development. However, for girder bridges, the prestressed strands are generally made of steel products with corrosion resistance and poor fatigue resistance. Due to the long-term high-stress state and environmental erosion, the steel strand often has fatigue damage or fatigue damage after several years of service, resulting in great economic losses [1]. Fiber-Reinforced Polymer (FRP) is made of high-performance fiber and resin matrix through a special production process. It has excellent properties of lightweight [2,3], high strength [4,5], corrosion resistance [6,7,8], and fatigue resistance [9,10]. In recent years, FRP has gradually attracted more popularity in the field of civil engineering [11,12,13]. Composite materials include carbon, glass, basalt, and aramid FRPs. Among them, CFRP is the most widely used and has been used in many fields [14,15]. CFRP not only has the advantages of FRP composites, but also has the characteristics of good creep resistance, extremely high tensile strength and specific strength, and its strength and alkali resistance are the highest among FRPs [8]. Compared to steel, under the same elastic modulus, the mass is only 1/5 of that of steel, and it is expected to replace traditional building materials, such as steel and concrete in bridge structures [16]. As one of the CFRP products, CFRP strands are used in bridges to replace original steel strands, which can effectively solve the problems that the original steel strands are prone to corrosion and fatigue damage, and greatly reduce unnecessary economic losses.

Nowadays, although the application research of CFRP bars and CFRP strands has made great progress, the anchoring problem has always been the main influencing factor that restricts the wide application of CFRP bars and CFRP strands. Nanni et al. [17] conducted performance tests on the FRP prestressed tendon anchoring system and proved that the anchoring performance of the anchoring system plays a decisive role in the performance of the prestressed FRP structure. Currently, the anchoring of CFRP bars and CFRP strands is divided mainly into two forms, namely the clip-type anchoring system and bonding anchoring system [18]. Furthermore, the wedge-bonding composite anchoring system that combines the clip and bonding anchorage together has also been proven to be an effective method to anchor the CFRP tendon with high anchoring efficiency [19]. Among them, the clip-type anchoring system is mainly composed of anchor rings and clips, which mainly use the friction force and mechanical occlusal performance of the anchor to anchor the CFRP prestressed tendons or prestressed strands. The disadvantage of the clip-type anchoring system is that the fatigue performance of this anchoring form is not good, resulting in damage to the CFRP prestressed tendons or prestressed strands. The bonding anchor system is mainly composed of the bonding medium and sleeve, and the CFRP prestressed tendons or prestressed strands are anchored by chemical adhesive force, friction force and mechanical bite force. Moreover, studies indicated that sand-coated can further improve the bonding performance of FRP [20,21]. Bonded anchor system has good fatigue resistance and is more suitable for the anchorage of CFRP prestressed tendons or prestressed strands.

At present, researchers carried out a series of research work on the anchorage of CFRP bars or prestressed strands. Tingjia Yu [22] studied the static test of the CFRP strand bonded anchorage, and the binder used was reactive powder concrete (RPC). The test results demonstrated that the anchoring form of CFRP strands in the anchoring area has a significant effect on the anchoring performance. Under the same conditions, the anchoring efficiency of the scattered anchoring is higher than that of the whole-bundle anchoring. Ronggui Liu [23,24] studied the design of CFRP strand clip-bonded tandem anchorage under static load test. The test results demonstrate that the load borne by the bonded anchor section is larger than that of the clip anchor section. In addition, the anchoring performance of the CFRP stay cable anchor was also studied, and the results demonstrated that the bonding length and bonding medium had a great influence on the anchoring performance of the anchor. Lili Sun [25] studied the internal mechanical behavior of the CFRP-reinforced anchorage. The results demonstrate that with the increase in the diameter of the CFRP bars or the radial compressive stress, the bearing capacity of the anchorage increases linearly, while the critical anchorage length decreases with the increase in the radial compressive stress. Puigvert et al. [26] used the finite element method to predict the fatigue performance and the creep performance of the bonded anchorage, and the results matched well with the test data. Zhang et al. [27] analyzed the working principle of the bonded anchorage, and conducted a pull-out test study on the FRP-reinforced ground anchorage poured with cement mortar. The results demonstrate that the surface morphology and the pouring material of the FRP reinforcement anchor section are the key factors affecting the anchorage performance. Suwei Hou [1] conducted experimental research on the anchorage mechanism of the CFRP cable-strand bonded anchors. The results demonstrate that the pre-tightening force and wire spacing have a great influence on the anchoring performance of the anchor, and a certain length of the anchor cylinder at the load end is reserved without pouring adhesive, which can solve the problem of CFRP wire bending near the load end. The German DSI company is where Noisternig [28] has developed an anchoring system named DYWICARB. The anchoring system is mainly composed of an inner cone-type steel sleeve and resin infusion materials. The static and dynamic tests have proved the reliability of the anchoring system. Qianhong Feng [29] studied the fatigue properties of CFRP tendon anchors, and the results demonstrated that the thicker the bonding medium, the more uniform the distribution of shear stress and compressive stress in the anchorage area. Tianyong et al. [26] and Chao et al. [30] studied the anchorage performance of the CFRP bar-bonded anchorage and the composite anchorage and proposed the main factors that affect the anchorage performance of the two anchorages. Meier et al. [31] proposed a new and reliable anchoring scheme, which uses advanced gradient materials based on ceramics and polymers to make variable-stiffness bonding media to anchor CFRP bars. It makes the shear stress distribution of the bonding medium contact surface more uniform. Sentry et al. [32] developed a bonding anchoring system for permanent anchors. Zhengyu Huang [33] conducted an experimental study on the bonding properties of activated powder concrete and carbon fiber reinforcement. The results demonstrate that the bond strength is related to the water-binder ratio and the amount of steel fibers, and the addition of silica fume can improve the bond properties of reactive powder concrete to some extent. Fang et al. [34] used reactive powder concrete as the bonding medium to anchor multiple CFRP bars. Na Zang [35] studied the CFRP bar bonding anchoring system using three bonding media: ordinary construction glue, Lica plant glue, and epoxy resin mortar. The test results demonstrate that the anchoring performance of epoxy mortar is the best, followed by Lica planting glue, and the anchoring performance of ordinary construction glue is the worst. Zhe Wang [36] carried out experimental research on the anchorage performance of CFRP-reinforced anchorage, used ANSYS to simulate and analyze the anchorage, and determined the influencing factors and failure modes. Dong Liang [37] conducted an experimental study on the static performance of carbon fiber prestressed tendons and cable anchorage systems. The test demonstrates that the rougher the surface of CFRP reinforcement, the higher the anchoring efficiency, and the ultimate load of the anchor with RPC as the bonding medium is the largest, which is better than epoxy mortar and ordinary concrete. Bujun et al. [38] proposed a single-bar anchoring device with simple fabrication and good performance, and performed uniaxial tensile tests on resin-coated CFRP bars, which proved that the anchoring device has good anchoring performance. Kuihua Mei et al. [39,40] previously conducted a static test study of the large-tonnage CFRP cable-stayed anchorage, and also applied the CFRP cable-stayed anchor to the first CFRP cable-stayed bridge in China. In summary, a large number of scholars at home and abroad have conducted in-depth research on the anchorage system of CFRP strands, including the design and verification of anchors, anchoring mechanism, etc., but there are few studies on the internal taper changes of CFRP prestressed strand anchors and the influence of different bonding media on the anchoring performance of anchors. On the basis of this, this paper will carry out the research on the performance of the bonded anchorage test of CFRP prestressed strands by considering those key parameters.

In this paper, the characteristics of CFRP material are fully considered, and it is made into a stranded wire; based on this, a bond-type anchorage test with better fatigue performance is carried out. First, the adhesively bonded anchors with better performance were developed through experimental work on the performance of the bonded CFRP prestressed strand by anchoring with different lengths under the condition of pouring the same adhesive medium. Then, the best-performing anchors were used to explore the influence of different bonding media such as epoxy resin, epoxy resin mortar, and ultra-high-performance concrete dry mix (UHPC-GJL) on anchorage performance. Finally, the suitable anchors with the appropriate bonding mediums for the conical anchor were developed.

## 2. Experimental Program

Firstly, the material properties tests of CFRP strands, epoxy resin, epoxy resin mortar, and UHPC-GJL are described in detail. Secondly, on the basis of understanding the performance parameters of each material, a comprehensive introduction is made to the prestressed strand bond type anchorage test process of carbon fiber-reinforced composite material, including the preparation of specimen, the layout of measuring points, and the loading scheme.

### 2.1. Material Test

#### 2.1.1. CFRP Strand

**Figure 1:**
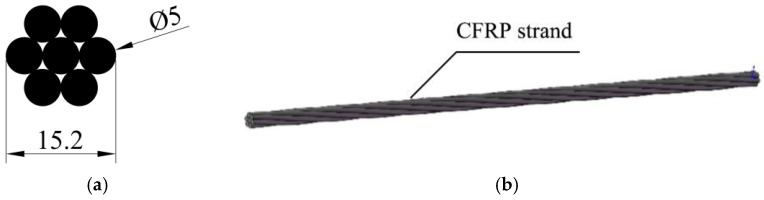
Cross-sectional dimensions of CFRP strands for testing. (**a**) CFRP strand section; (**b**) CFRP strand for testing.

The prestressed tendons used in this test are CFRP strands with nominal diameter of 15.2 mm produced by Jiangsu Lianyungang Zhongfucarbon-core Cable Technology Co., Ltd (Lianyungang, China). The effective cross-sectional area of the CFRP strands are 137.44 mm^2^. The mono-wire is produced by the pultruded molding process, and the CFRP strands are made of 7 wires through continuous carbon fiber mono-wire dipping, twisting, molding, and curing. The material property test results of CFRP strands are provided by the manufacturer. The cross-sectional dimensions and physical properties of the CFRP strands used in the test are shown in Figure 1 and Table 1.

#### 2.1.2. Bonding Medium

**Figure 2:**
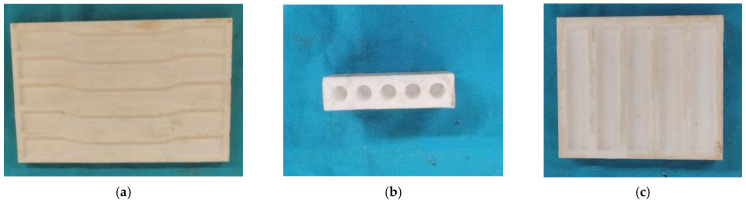
Epoxy resin and epoxy resin mortar specimen molds. (**a**) Tensile specimen mold; (**b**) compression specimen mold; (**c**) bending specimen mold.

For the conical anchor, the bonding medium is subjected to a large hoop pressure during the loading process of the specimen. Therefore, when selecting the bonding medium, it is necessary to consider not only the bonding performance of the bonding medium, but also its stiffness and interface bonding effect. Therefore, in this experiment, three bonding media with different stiffness and bonding performance are used as the pouring material of the anchor. The three bonding media used in this experiment are epoxy resin, epoxy resin mortar, and ultra-high-performance concrete dry mix (UHPC-GJL). The epoxy resin adopts Miuhan’s carbon fiber-reinforced glue, which is composed of A glue and B glue. The A glue and the B glue are evenly stirred at a mass ratio of 2:1 to make the adhesive. The epoxy resin mortar is stirred uniformly by epoxy resin and quartz sand in a mass ratio of 2.5:1. The size of the quartz sand particles is divided into two types, 40–80 mesh and 120–180 mesh, and the ratio is 1:2. The UHPC-GJL is an ultra-high-performance concrete dry mix produced by Hunan Changsha Guli Engineering New Materials Co., Ltd., in which the water and dry mix are uniformly stirred in a mass ratio of 9.5% to make a grouting material. According to the GB/T2567-2008 specification [41] of the performance test method for the resin cast body, standard molds are made of poly tetrafluoroethylene for tensile, compression, and bending specimens, as shown in Figure 2.

**Figure 3:**
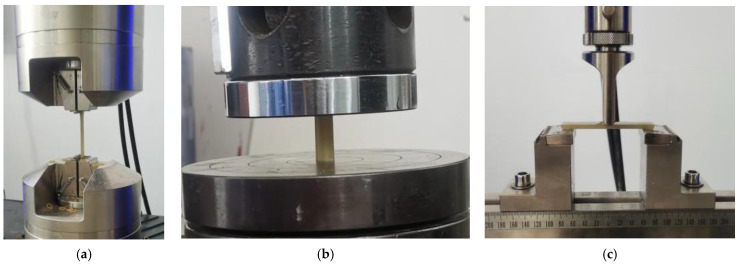
Epoxy resin and epoxy resin mortar material property test. (**a**) Tensile test; (**b**) compression test; (**c**) bending test.

In this study, five specimens are made for each epoxy resin and epoxy resin mortar bonding medium using standard molds. After curing to reach its strength, the tensile, compressive and bending tests are carried out on each specimen. The test process and results are shown in Figure 3 and Table 2.

**Figure 4:**
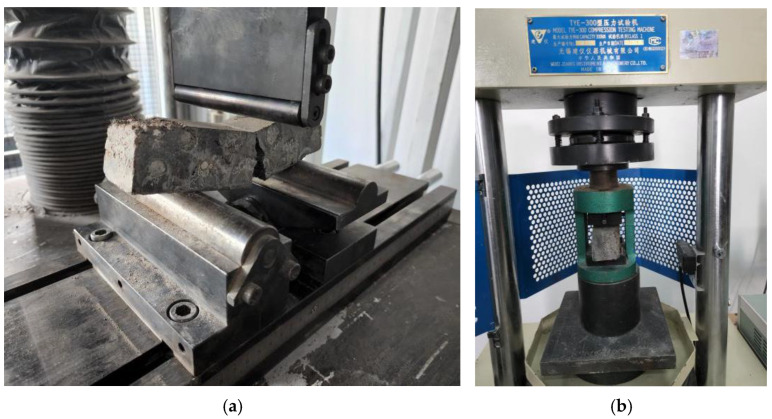
UHPC-GJL test block material property test. (**a**) Breaking test; (**b**) compression test.

Furthermore, according to the specification GB/T50448 for cement-based grinding materials [42], a 40 mm × 40 mm × 160 mm UHPC-GJL prismatic test block was made and the compression test was carried out after curing. The test process and material parameters are shown in Figure 4 and Table 3.

### 2.2. Anchorage Test

#### 2.2.1. Specimen Preparation

**Figure 5:**
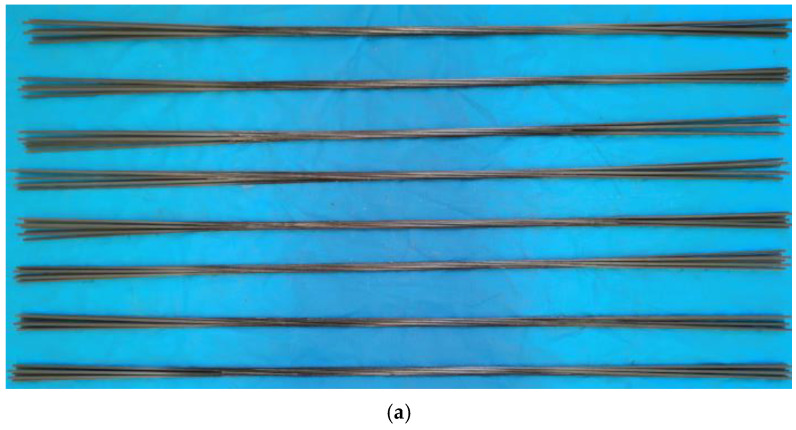

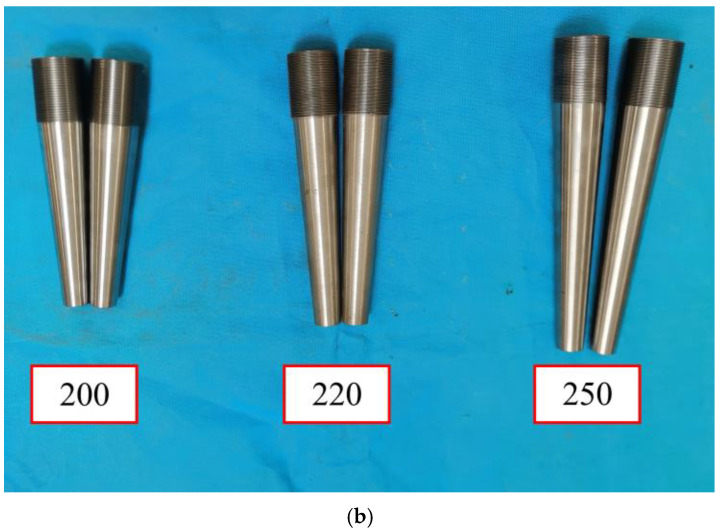
Partial CFRP strand and conical anchors for testing. (**a**) Partial CFRP strand for testing; (**b**) conical anchors for testing.

In this paper, a total of six groups of the experiment were designed, namely the anchor control group and the adhesive medium control group, and the number of specimens in each group was 2. Among them, the anchor control group was set up with three groups of 200 mm, 220 mm, and 250 mm to evaluate the influence of the increase in the length of the anchor; the inner taper decreased in turn, and the bonding medium was epoxy resin. The bonding medium control group set up again with three groups of bonding mediums such as epoxy resin, epoxy resin mortar, and UHPC-GJL. In order to facilitate the statistics of the test results, the specimen numbers of the anchor control group are 200-X, 220-X, and 250-X, and the adhesive medium control group are E-X, EM-X, and GJL-X, where X represents the number of the specimen. The test specimen in this paper is composed of CFRP strand and two anchors, which are shown in Figure 5.

**Figure 6:**
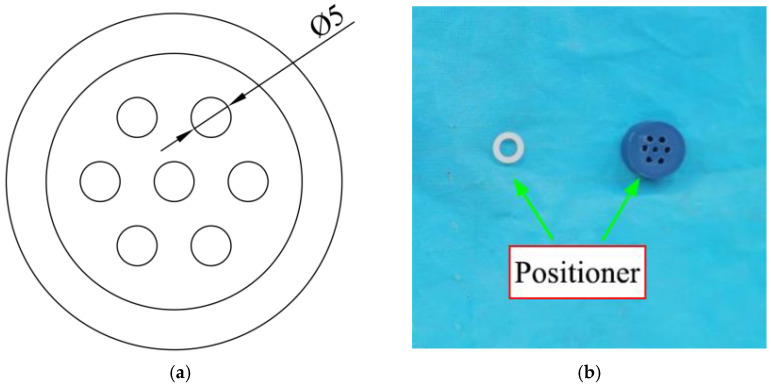
CFRP strand positioning device. (**a**) Locator design; (**b**) physical map of the locator.

**Figure 7:**
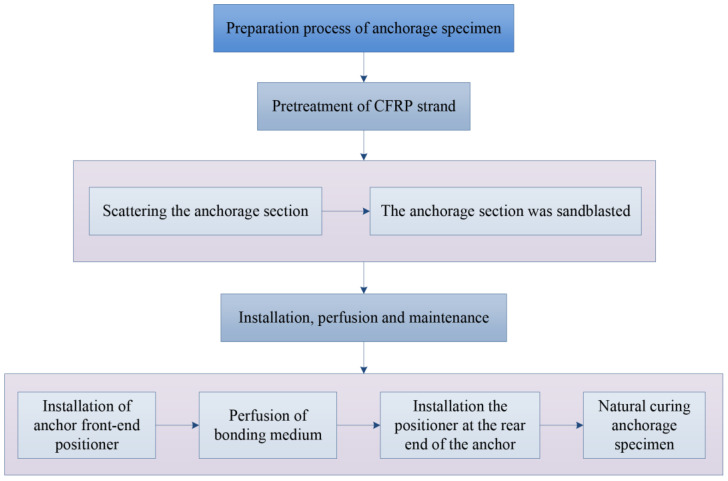
Flow diagram of CFRP strand anchorage process.

In the process of making the specimens in this test, the CFRP strands were first treated, including the scattered anchoring section and the surface sandblasting of the anchoring section, so as to increase the anchorage area and roughness of the CFRP strands. The locator is then designed, as shown in Figure 6, to ensure that the CFRP strands are uniformly stressed in the anchoring section and to improve the anchoring performance of the tapered anchorage. After completing the above preparations, the installation and perfusion of the specimen are processed. During the perfusion process, one-time perfusion should be guaranteed and maintained for a specified period of time to ensure that it reaches the specified strength. The flow diagram of anchorage process is shown in Figure 7.

#### 2.2.2. Measuring Point Layout and Loading Scheme

**Figure 8:**
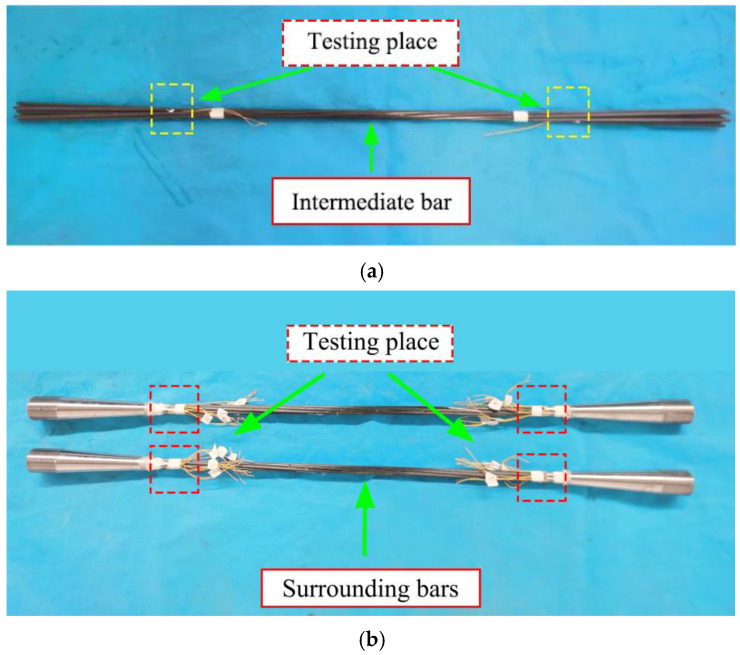
CFRP stranded wire measuring point layout. (**a**) Layout of measuring points in middle wire; (**b**) layout of the measurement points on each side wire.

To better study the stress and strain of the CFRP strand during the tensioning process, two strain measuring points were arranged on each CFRP strand wire in the experiment. The position of the measuring point was about 3 mm from the anchorage outlet. Among them, the layout of the measurement points of the middle wire is completed before the bonding medium is poured. The measurement points of each side wire are arranged after the specimen maintenance is complete, as shown in Figure 8.

**Figure 9:**
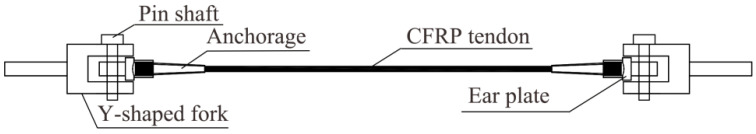
Assembly drawing of the CFRP strand tensile specimen.

**Figure 10:**
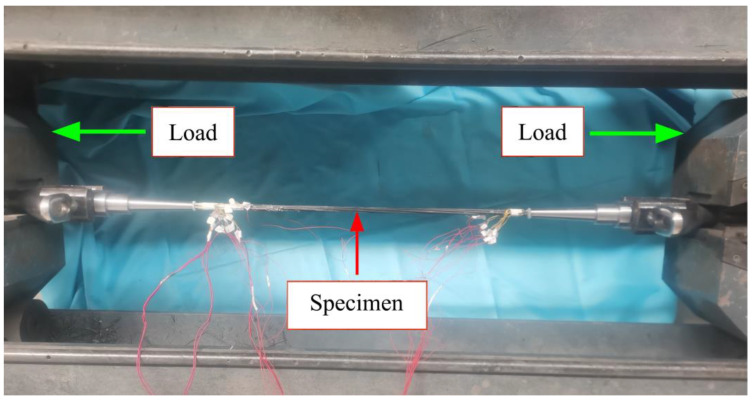
Specimen loading diagram.

The test adopts the horizontal tensile testing machine with a maximum tensile force capacity of 600 kN of Zhongfu Carbon Core Cable Technology Co., Ltd (Lianyungang, China). In order to avoid additional torque of the specimen during the loading process, the specimen is fixed in the form of hinges. The chuck of the testing machine clamps the Y-shaped tooling, the Y-shaped tooling is connected with the ear plate through the pin shaft, and the ear plate is connected with the conical anchor through the thread, as shown in Figure 9 after assembly. According to the specification recommendations, this test adopts the displacement loading method, and the loading speed is 1.5 mm/min. In addition, the strain gauges are connected with the dynamic strain acquisition instrument by wire, and the acquisition instrument monitors the strain of the specimen in real time. The loading diagram of the specimen is shown in Figure 10.

## 3. Experimental Results and Discussions

The statistical analysis of the experimental results along with detailed discussions of experimental findings on the bonded anchorage of CFRP prestressed strands have been presented in this section. Firstly, the failure modes, the anchoring efficiency coefficient of the anchors as well as load-displacement relationship curves for all groups of specimens have been presented and discussed in the Section 3.1. Then, Section 3.2 will comprehensively analyze the stress–strain results of the two groups of specimens E-X and EM-X, and discuss the stress and failure characteristics of each CFRP strand under the condition of using different bonding media. Furthermore, Section 3.3 mainly expounds the influence of the anchorage taper on the anchorage performance, and then proposes a more reasonable anchorage size. Finally, the influence of the bonding medium on the performance of the anchor has been analyzed in the Section 3.4 and proposes a more suitable bonding medium for the conical anchor.

### 3.1. Form of Destruction

The detailed statistics and discussions on the test results of each group of specimens, including the failure modes of the specimens and the anchoring efficiency coefficient of the anchors, will be presented in this section. All the anchors met the requirements of the test, as none of the anchors were damaged and the stress was good during the testing. Therefore, the CFRP strand and the anchoring system are more fragile and their failure form will directly reflect the advantages and disadvantages of the corresponding anchoring system or bonding medium, as well as CFRP strands. Based on this, this paper will focus on analyzing the stress state and failure form of CFRP strands and anchoring systems.

**Figure 11:**
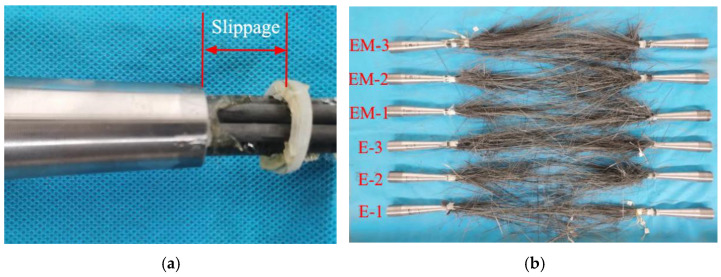
The damage form of CFRP strand. (**a**) CFRP strand pull-out failure; (**b**) explosive destruction of CFRP strand.

There are two failure modes of the specimens that have been observed during the experimental study. One is that the anchoring system failed to anchor the CFRP strand reliably. When the tension reached at a certain value, the CFRP strand is pulled out. The other is that the CFRP strand is anchored more reliably by the anchoring system, and the CFRP strand is explosively damaged in the free section. The failure modes of the specimen are shown in Figure 11.

In the anchor control group, all the four specimens of 200 mm anchor length suffered explosive damage of the CFRP strand. In contrast, all the specimens of 220 mm anchors’ length were failed by CFRP strand pull-out. Meanwhile, the 250-1 specimens of the 250 mm anchorage suffered explosive damage of CFRP strand, and 250-2 suffered from CFRP strand pull-out damage. In the adhesive medium control group, the anchors used were 200 mm anchor length. When the bonding medium was poured with epoxy resin and epoxy resin mortar, all specimens suffered an explosive failure of the CFRP strand. While the specimens poured UHPC-GJL, all the specimens suffered from CFRP strand pull-out failure. The statistical results and the failure modes of anchor control specimens and adhesive medium control specimens are shown in Table 4 and Table 5, respectively. Among them, the anchoring efficiency coefficient mentioned in this section reflects the pros and cons of the anchoring performance of the anchoring system in different test groups, and the anchoring efficiency coefficient = actual ultimate load/manufacturer’s standard ultimate strength × 100%.

It can be observed from Table 4 that in the anchoring control group, the anchoring efficiency of the 200 mm anchor is the best, the lowest anchoring efficiency is 91.8%, the average anchoring efficiency is 96.4%, and the variance is the smallest, which is 18.8(%)^2^. It shows that the anchor can reliably anchor CFRP strands when pouring epoxy resin. Compared with 200 mm anchors, the minimum anchoring efficiencies of 220 mm and 250 mm anchors were 83.6% and 87.7%, respectively, and the average anchoring efficiencies were 92.7% and 93.8%, both lower than 95%. The variances are 81.9(%)^2^ and 36.6(%)^2^, respectively; the variance is large and the data is more discrete. It shows that the anchoring performance of the above two anchors is not very reliable. Therefore, in the follow-up adhesive medium control group, the 200 mm anchor with better anchoring performance will be used.

It can be observed from Table 5 that in the bonding medium control group, the anchoring efficiency of the 200 mm anchor with epoxy resin mortar is the highest, the lowest is 92.9%, the highest is 95.1%, and the variance is 1.2(%)^2^. The anchoring efficiency of the 200 mm anchors poured with epoxy resin is higher, the lowest anchoring efficiency is 91.8%, the highest is 102.8%, and the variance is 18.8(%)^2^. However, the anchoring performance of the 200 mm anchors filled with UHPC-GJL is the worst, and the anchoring efficiency is lower than 50%. This kind of bonding medium cannot reliably anchor the CFRP strands.

**Figure 12:**
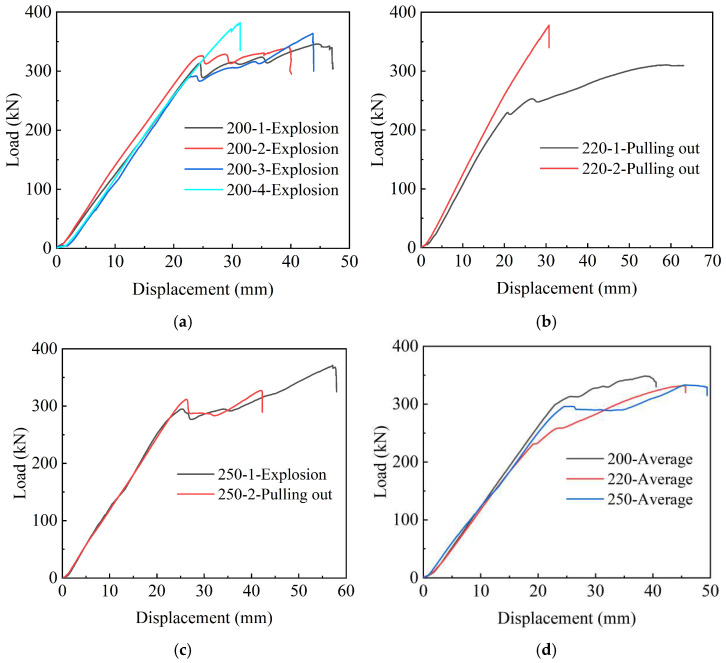
Force-displacement relationship curves of anchor control group. (**a**) Force-displacement curves of 200-X test group; (**b**) force-displacement curves of 220-X test group; (**c**) force-displacement curves of 250-X test group; (**d**) average of force-displacement curves.

**Figure 13:**
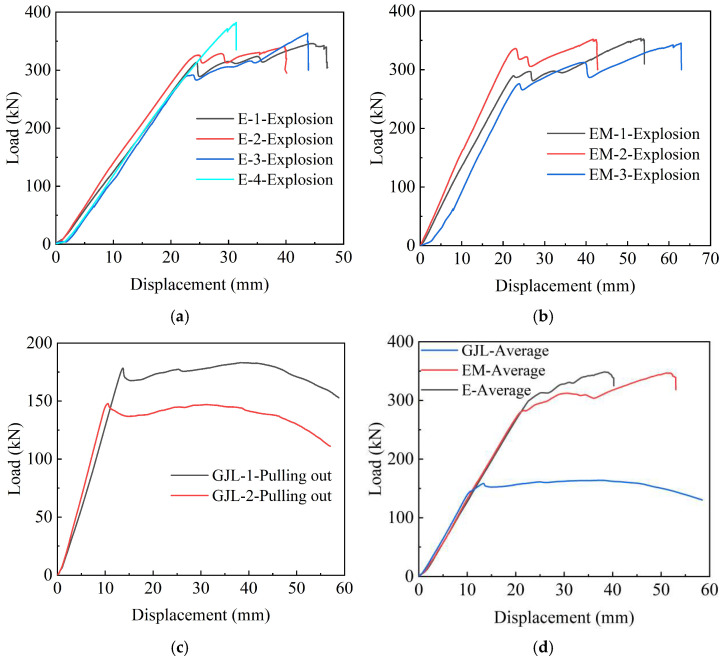
Force-displacement relationship curves of bonding medium control group. (**a**) Force-displacement curves of E-X test group; (**b**) force-displacement curves of EM-X test group; (**c**) force-displacement curves of GJL-X test group; (**d**) average force-displacement curves.

In order to more intuitively demonstrate the loading process of each specimen in the two control groups, the force–displacement relationship curves of the two control groups are drawn and presented in Figure 12 and Figure 13. It can be observed from Figure 12a–c that all the three 200 mm, 220 mm, and 250 mm anchors are shown with linear characteristics in the initial stage. Hence, the bonding medium in the anchorage can reliably anchor the CFRP strand under the combined action of the hoop pressure of the anchorage, the bonding force between the anchorage, and the bonding medium and the frictional force. When the tensile load increased to a certain value, most of the curves fluctuated because the epoxy resin in the anchor had a small deformation under the influence of pressure, resulting in a stress redistribution. In addition, a small part of the curve basically maintains the linear characteristics, and there is no large curve fluctuation, such as the two specimens, 200-4 and 220-2. It can be observed from Figure 12d that the average anchoring performance of the 200 mm anchor is better than that of the 220 mm and 250 mm anchors.

In order to more intuitively show the loading process of each specimen in the two control groups, the force-displacement relationship curves of the two control groups are drawn and presented in Figure 12 and Figure 13.

It can be seen from Figure 12a–c that all the three 200 mm, 220 mm and 250 mm anchors are shown linear characteristics in the initial stage. Hence, the bonding medium in the anchorage can reliably anchor the CFRP strand under the combined action of the hoop pressure of the anchorage, the bonding force between the anchorage and the bonding medium and the frictional force. When the tensile load increased to a certain value, most of the curves fluctuated because the epoxy resin in the anchor had a small deformation under the influence of pressure, resulting in a stress redistribution. In addition, a small part of the curve basically maintains the linear characteristics, and there is no large curve fluctuation, such as the two specimens 200-4 and 220-2. It can be seen from Figure 12d that the average anchoring performance of the 200 mm anchor is better than that of the 220 mm and 250 mm anchors.

It can be observed from Figure 13a–c that under the same anchoring conditions, the force-displacement curves of the specimens poured with epoxy resin and epoxy resin mortar are basically the same as described above. The curves of the two groups of specimens are linear in the early stage and fluctuating in the middle stage. When a certain load is reached, explosive failure occurs. For the specimens poured with UHPC-GJL, the ultimate bearing capacity is lower, and all are less than 200 kN. It can be observed from Figure 13d that the anchoring performance of the specimen poured with epoxy resin and epoxy resin mortar is better, while the specimen poured with UHPC-GJL has greater slippage and poor anchoring performance.

### 3.2. Stress–Strain Relationship

**Figure 14:**
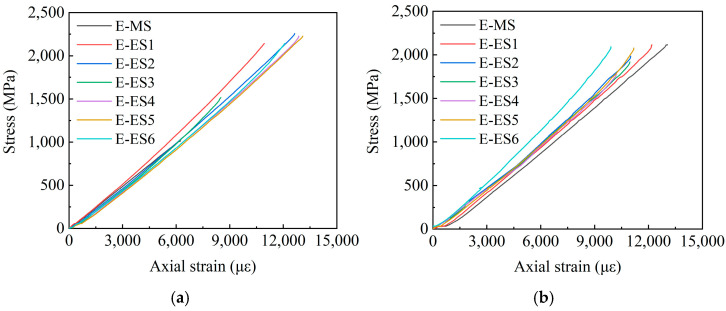
E-X group stress–strain curve. (**a**) E-1 stress–strain curve; (**b**) E-3 stress–strain curve.

**Figure 15:**
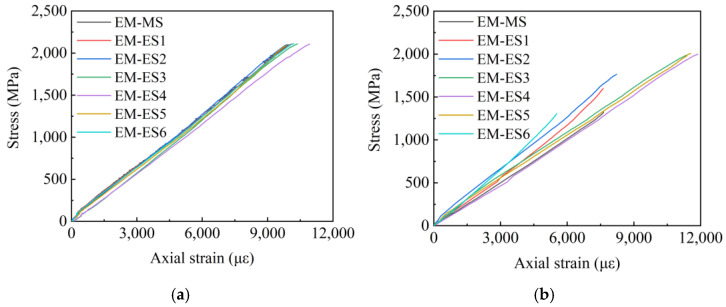
EM-X group stress–strain curve. (**a**) EM-1 stress–strain curve; (**b**) EM-2 stress–strain curve.

**Figure 16:**
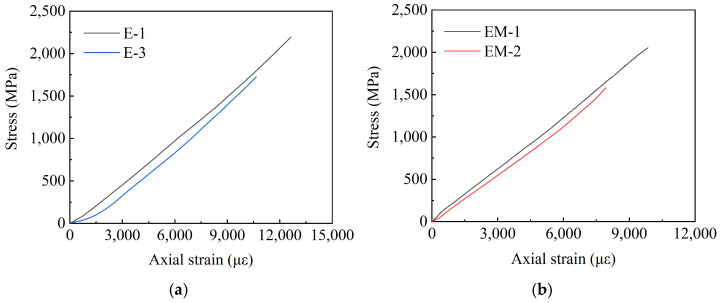
Average stress–strain curves of E-X and EM-X test groups. (**a**) E-X average stress–strain curve; (**b**)EM-X average stress–strain curve.

According to Section 3.1, the main explosive failures are in the E-X and EM-X groups; thus, this section will draw the stress–strain curves of the above two groups of specimens. Given that the acquisition of stress–strain data of the three specimens, E-2, E-4, and EM-3 failed during the test, the following figures will present the stress–strain curves of the remaining specimens. Among them, E-MS and EM-MS represent the middle wire stress–strain curves of the two groups of specimens, and E-ES and EM-ES represent the edge wire stress–strain curves of the two groups of specimens. Figure 14 shows the E-X stress–strain curve, Figure 15 shows the EM-X stress–strain curve, and Figure 16 shows the E-X and EM-X average stress–strain curves.

It can be observed from Figure 14 and Figure 15 that in the adhesive medium control group, the strain of the middle filaments of the E-X and EM-X groups of specimens increased rapidly, and the strain gauge appeared prematurely failed. During the loading process of the specimen, the stress of the middle wire increases rapidly, and the edge wire squeezes the middle wire. In addition, the stress–strain curve of each wire of the E-X test group is relatively scattered, indicating that the force of each wire of the specimen is not uniform. For the EM-X test group, the dispersion of the stress–strain curves of each wire is small, it is always consistent before the peak value, and the force of each wire of the CFRP strand is relatively uniform, indicating that the overall performance of the EM-X specimen is better than that of the E-X specimen. The reason is that adding an appropriate amount of quartz sand helps to increase the comprehensive performance of epoxy resin, thereby improving the anchoring performance of the anchor. It can be observed in Figure 15 that the average stress–strain curves of the two groups of specimens are approximately linear before the peak point, indicating that the specimens exhibit linear elastic characteristics before failure, which is a typical brittle failure mode.

It can be seen from Figure 14 and Figure 15 that in the adhesive medium control group, the strain of the middle filaments of the E-X and EM-X groups of specimens increased rapidly, and the strain gauge appeared premature failure. During the loading process of the specimen, the stress of the middle wire increases rapidly, and the edge wire squeezes the middle wire. In addition, the stress-strain curve of each wire of the E-X test group is relatively scattered, indicating that the force of each wire of the specimen is not uniform. For the EM-X test group, the dispersion of the stress-strain curves of each wire is small, and it is always consistent before the peak value, and the force of each wire of the CFRP strand is relatively uniform, indicating that the overall performance of the EM-X specimen is better than that of the E-X specimen. The reason is that adding an appropriate amount of quartz sand helps to increase the comprehensive performance of epoxy resin, thereby improving the anchoring performance of the anchor. It can be seen in Figure 16 that the average stress-strain curves of the two groups of specimens are approximately linear before the peak point, indicating that the specimens exhibit linear elastic characteristics before failure, which is a typical brittle failure mode.

In summary, both epoxy resin and epoxy resin mortar bonding medium can anchor CFRP strand reliably, where the anchor with epoxy resin medium has demonstrated the highest anchoring efficiency, and the anchor with epoxy resin mortar has demonstrated the overall anchoring performance better than that of the anchor with the epoxy resin bonding medium.

### 3.3. Influence of Anchor Taper

**Figure 17:**
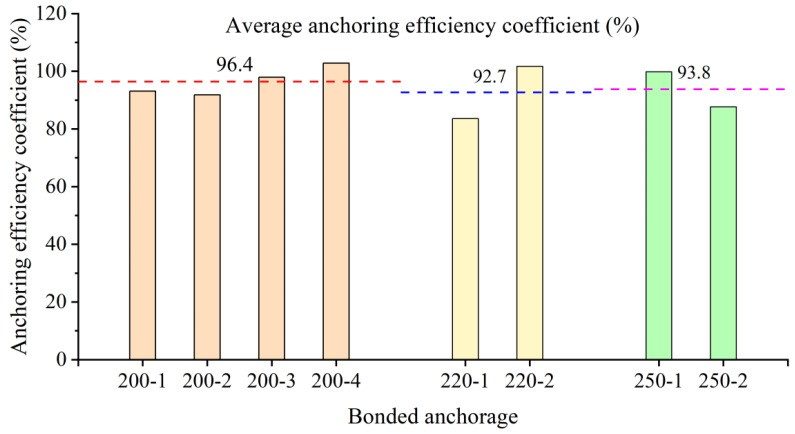
Comparison chart of anchoring efficiency coefficients of three types of anchors.

In this paper, the anchoring performance of tapered anchors with lengths of 200 mm, 220 mm, and 250 mm is experimentally studied and compared. The inner diameters of the three lengths of anchors are the same, and the taper shows a decreasing trend, which are 2.29°, 2.08°, and 1.83°, respectively. The anchoring efficiency coefficients of the three anchors are shown in Figure 16. It can be observed from Figure 17 that the anchor performance of the 200 mm anchor is better, as the anchoring efficiency is higher, and the average anchoring efficiency coefficient is 96.4%. The average anchoring efficiencies of the 220 mm and 250 mm anchors are 92.7% and 93.8%, respectively, and both are shown to have 3.7% and 2.6% less anchoring efficiency than the 200 mm anchor. In addition, in the 220 mm anchorage and the 250 mm anchorage, the difference between the anchoring efficiency coefficients of each specimen is large, indicating that the above two anchorages have comparatively poor anchorage performance. It can be observed that even if the length of the anchor section is increased, if the taper of the anchor is reduced, the anchoring performance will be greatly affected. It has been proved that the taper is the key factor affecting the anchor.

### 3.4. Influence of Bonding Medium

**Figure 18:**
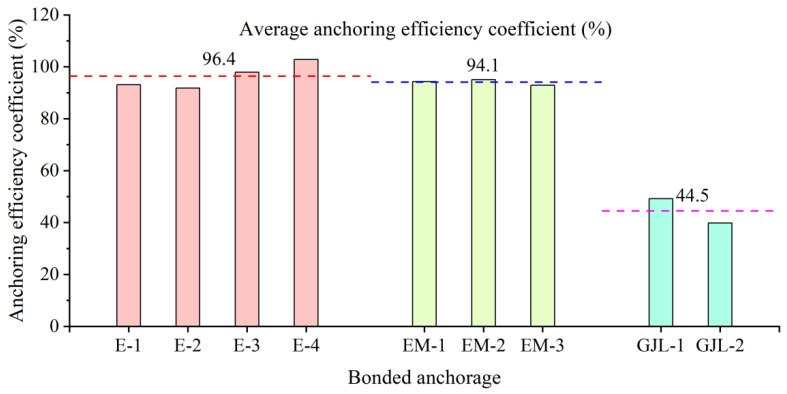
Comparison of anchoring efficiency coefficients of different bonding medium.

In order to verify the influence of the bonding medium on the anchoring performance of the conical anchor, this paper uses the 200 mm anchor, as it has a better anchoring performance to conduct a comparative study of the bonding medium performance. It can be observed from Figure 18 that all the specimens with the epoxy resin bonding medium are explosively damaged, the anchoring performance of the anchor is relatively stable, the average anchoring efficiency is higher, and its value is 96.4%. It demonstrates that for the 200 mm tapered anchor, epoxy resin can reliably anchor CFRP strands. When a certain amount of quartz sand is mixed into the epoxy resin, the average anchoring efficiency of the anchor is 94.1%, which indicated that adding an appropriate amount of quartz sand into the epoxy resin is helpful to improve the stress of the CFRP strand. For the specimen infused with the UHPC-GJL bonding medium, the anchoring performance of the anchor is the worst, and the CFRP strands are all pulled out, and the average anchoring efficiency coefficient is the lowest, at only 44.5%. It demonstrates that for the 200 mm tapered anchors, the UHPC-GJL bonding medium cannot reliably anchor CFRP strands, because the coupling between this bonding medium and CFRP material is not good—the two cannot be well bonded.

## 4. Conclusions

A detailed experimental study has been conducted to investigate the performance of the bonded anchorage of CFRP prestressed strands. The influences of different anchor length and bonding media on the anchorage performance have been performed experimentally to find out the most appropriate bonded anchorage of CFRP prestressed strands. The following conclusions can be drawn based on the comparative analyses and discussions of the experimental results:

- The bonded anchorage of CFRP prestressed strand with 200 mm anchor is most efficient in this study, as the taper of the 200 mm anchor is the largest. The average anchoring efficiency coefficient of the 200 mm anchor was 96.4%, which is 3.7% and 2.6% higher than the average anchoring efficiency coefficient of 220 mm and 250 mm anchors, respectively. This shows that the taper is the key factor affecting the anchoring performance of this kind of anchor.
- The bonded anchorage of the CFRP strand with a 200 mm anchor is the most reliable, as all the specimens of the 200 mm anchor demonstrated less difference of anchoring efficiencies and exhibited consistency in the failure mode by demonstrating the explosive failure of the CFRP stand. On the contrary, the difference in anchoring efficiencies between the specimens of 220 mm and 250 mm anchors is quite large, at 18.1% and 12.1%, respectively.
- When epoxy resin mortar is used as the bonding medium of the 200 mm anchor, the anchoring efficiency is also higher, with less difference in anchoring efficiencies between the specimens; all CFRP strands are explosively damaged. Adding an appropriate amount of quartz sand into the epoxy resin helps to improve the comprehensive performance of the bonding medium, and to a certain extent, improves the overall anchoring performance of the anchor and the stress of the CFRP strand as the dispersion of the stress–strain curves of each wire for the anchor with epoxy resin mortar medium is small, and it is always consistent before the peak value; the force of each wire of the CFRP strand is relatively uniform.
- Both the epoxy resin and epoxy resin mortar bonding medium can anchor the CFRP strand reliably, where the anchor with the epoxy resin medium has demonstrated the highest anchoring efficiency, and the anchor with epoxy resin mortar has shown the overall anchoring performance better than that of the anchor with the epoxy resin bonding medium.
- The bonded anchorage of the CFRP prestressed strand with infused with UHPC-GJL bonding medium and the anchoring performance of the anchor is the worst; the CFRP strands are all pulled out, and the average anchoring efficiency coefficient is the lowest, at only 44.5%. Therefore, the UHPC-GJL bonding medium cannot reliably anchor CFRP strands, because the coupling between this bonding medium and the CFRP material is not good, and the two cannot be well bonded.

## Figures and Tables

**Table 1:** CFRP strand material properties.

Material Properties	Average Tensile Strength/ MPa	Elastic Modulus/ GPa	Density/ (kg·m^−3^)	Ultimate Elongation %	Fiber Volume Fraction %
CFRP strand	2703.78	170	1.65	1.50	71

**Table 2:** Performance parameters of epoxy resin and epoxy resin mortar material.

Material Properties	Average Tensile Strength/ MPa	Average Compressive Strength/ MPa	Average Bending Strength/ MPa
Epoxy resin	63.66	88.36	141.64
Epoxy resin mortar	33.20	85.94	60.95

**Table 3:** UHPC-GJL material performance parameters.

Material Properties	Average Breaking Strength/ MPa	Average Compressive Strength/ MPa
UHPC-GJL	33.37	168.50

**Table 4:** Statistical results and the failure modes of anchor control specimens.

Group Name	Adhesive Medium	Specimen Number	Specimen Length [mm]	Free Segment Length [mm]	Form of Destruction	Factory Standard Ultimate Load [kN]	Actual Ultimate Load [kN]	Anchoring Efficiency Coefficient *μ* [%]	Anchoring Efficiency Coefficient Variance $\mu^{2}\left[{\left(\%\right)}^{2}\right]$
200-X	Epoxy resin	200-1	1100	700	Explosion	371.6	345.8	93.1	18.8
		200-2	1100	700	Explosion	371.6	341.3	91.8	
		200-3	1100	700	Explosion	371.6	363.8	97.9	
		200-4	1100	700	Explosion	371.6	382.0	102.8	
220-X	Epoxy resin	220-1	1140	700	Pulling out	371.6	310.6	83.6	81.9
		220-2	1140	700	Pulling out	371.6	378.0	101.7	
250-X	Epoxy resin	250-1	1200	700	Explosion	371.6	371.0	99.8	36.6
		250-2	1200	700	Pulling out	371.6	325.8	87.7	

**Table 5:** Statistical results of the failure modes of the specimens in the control group with the bonding medium.

Group Name	Adhesive Medium	Specimen Number	Specimen Length [mm]	Free Segment Length [mm]	Form of Destruction	Factory Standard Ultimate Load [kN]	Actual Ultimate Load [kN]	Anchoring Efficiency Coefficient *μ* [%]	Anchoring Efficiency Coefficient Variance $\mu^{2}\left[{\left(\%\right)}^{2}\right]$
E-X	Epoxy resin	200-1	1100	700	Explosion	371.6	345.8	93.1	18.8
		200-2	1100	700	Explosion	371.6	341.3	91.8	
		200-3	1100	700	Explosion	371.6	363.8	97.9	
		200-4	1100	700	Explosion	371.6	382.0	102.8	
EM-X	Epoxy resin mortar	EM-1	1100	700	Explosion	371.6	350.6	94.3	1.2
		EM-2	1100	700	Explosion	371.6	353.4	95.1	
		EM-3	1100	700	Explosion	371.6	345.3	92.9	
GJL-X	UHPC-GJL	GJL-1	1100	700	Pulling out	371.6	183.0	49.2	22.1
		GJL-2	1100	700	Pulling out	371.6	147.9	39.8	

## Data Availability

Data is contained within the article.
